# Whole body gestational donation

**DOI:** 10.1007/s11017-022-09599-8

**Published:** 2022-11-18

**Authors:** Anna Smajdor

**Affiliations:** University of Oslo, Oslo, Norway

**Keywords:** Pregnancy, Surrogacy, Organ donation, Brain death, Procreation, Reproduction, Morality

## Abstract

Whole body gestational donation offers an alternative means of gestation for prospective parents who wish to have children but cannot, or prefer not to, gestate. It seems plausible that some people would be prepared to consider donating their whole bodies for gestational purposes just as some people donate parts of their bodies for organ donation. We already know that pregnancies can be successfully carried to term in brain-dead women. There is no obvious medical reason why initiating such pregnancies would not be possible. In this paper, I explore the ethics of whole-body gestational donation. I consider a number of potential counter-arguments, including the fact that such donations are not life-saving and that they may reify the female reproductive body. I suggest if we are happy to accept organ donation in general, the issues raised by whole-body gestational donation are differences of degree rather than substantive new concerns. In addition, I identify some intriguing possibilities, including the use of male bodies–perhaps thereby circumventing some potential feminist objections.

## Introduction

In 2000, Rosalie Ber advanced a novel suggestion for circumventing the moral problems of gestational surrogacy. She proposed that female patients in a persistent vegetative state (PVS) who had given prior written consent, could function as surrogates: embryos would be placed in the woman’s uterus and gestated to term [1]. To my knowledge, no jurisdiction has considered implementing Ber’s suggestion. This is surprising, given the degree to which surrogacy continues to provoke moral and legal controversy [2–5].

Ber does not give a name to the phenomenon she proposes; for the purposes of this paper, I use the term ‘whole body gestational donation’ (WBGD). Although my initial interest in this area was sparked by Ber’s work, I have adapted and extended her approach in three important ways that have both practical and normative implications, meaning that WBGD in my usage is not precisely the same phenomenon that she discusses in her paper.

- Ber’s view is that women in PVS would offer the best alternative to living surrogacy. I suggest that we should be willing to consider WBGD in patients who are brain stem dead rather than in PVS (and would therefore be eligible to be organ donors).
- Ber believes that WBGD should be available only to the patients deemed to have a clear medical need for it. I suggest that – all other things being equal–it should be an option for anyone who wishes to avoid the risks and burdens of gestating a foetus in their own body.
- Ber implicitly accepts that only women can gestate. I suggest that brain stem dead men would also have the potential to gestate, meaning that the pool of potential donors is further increased – and that certain feminist concerns might thus be assuaged.

In what follows, I will show why my adapted and extended version of WBGD offers a solution to the problems of surrogacy. I suggest that states and health services should adapt their policies and procedures to allow for WBGD among other donation options. I address some possible objections and show that although the prospect of WBGD may be disconcerting, most of the ethical problems that might be associated with it apply equally to other areas of medical and/or reproductive practice.

## PVS versus brain stem death

There is increasing evidence to confirm the fact that pregnancies can be carried to term in women who have suffered brain haemorrhages or other medical problems that have resulted in brain death [6–10]. Likewise, pregnancies have been recorded in women in a PVS [11]. Since both PVS and brain death are compatible with gestation, it is worth considering why Ber focussed on PVS in her original discussion. She does not provide much detail on this but does note the requirement of ventilation for brain-dead patients. Ventilation is resource-intensive and complex; patients who do not require it will therefore be easier to manage in at least some respects and will almost certainly cost less. This in itself might seem to give PVS a prima facie advantage over brain death as a source of patients to undertake gestation.

Interestingly, however, Ber urges that brain death should be *redefined* specifically so that PVS patients can be included in that category, and thus, to engage in gestational donation. However, this alone would be a complex undertaking. It would also have implications beyond gestational donation. If the criteria for brain death are changed, it would suggest that PVS patients might be eligible not just for gestational donation but for other forms of organ donation. This in turn might aggravate existing disputes and concerns about the concept of brain stem death [12]. Although brain stem death diagnosis has its critics, it is largely accepted by medical professionals. However, PVS diagnoses have been repeatedly called into question, especially in terms of their prognosis [13–15].

For this reason, if there is a way of achieving the benefits that Ber identifies *without* redefining brain death, this would be preferable. There are a number of additional reasons to support this. Firstly, PVS is a more unusual phenomenon than brain death. Willemijn Van Erp et al. suggest a prevalence of 0.1 to 0.2 PVS type patients per 100,000 members of the general population [15]. This equates to 0.01–0.02 per million. In contrast, the UK’s National Health Service gives figures of 18 cases of brain stem death per million of the general population [16]. Secondly, although there are those who dispute their validity, the use of brain stem death criteria for determining when a patient’s life is effectively at an end is widespread in the context of organ donation. In contrast, it is not so clear that PVS patients’ living interests are at an end; they *may* recover fully or partially. Patients who are brain stem dead *cannot* recover. Irreversibility is written into the definition of brain death. Accordingly, a patient who recovers was never *really* brain-dead in the first place. It is this that makes brain stem death the preferred route to organ donation.

However, before moving on to discuss other aspects of WBGD, there are some further issues related to brain death more generally that require consideration. Firstly, as suggested, the phenomenon of brain death itself and its role in facilitating organ transplantation is the subject of some criticism and indeed scepticism [17]. I acknowledge this debate and share some of the scepticism. But for the purposes of my argument, I need not enter this dispute. Those who accept brain stem death as an adequate basis for organ donation, should for consistency acknowledge its acceptability for WBGD as well. For those who reject the brain stem death criteria, clearly both organ donation and WBGD will be problematic.

## Consent

Ber insists that written explicit consent would be necessary from donors undergoing surrogacy in PVS. However, given that a patient in PVS cannot give informed consent, this would entail that people give consent for WBGD in PVS in advance of PVS happening to them. I have observed that PVS is a rare phenomenon. In practical terms, requiring consent from women prior to PVS surrogacy means that a woman must (a) have thought about the prospect of PVS and (b) decided to proactively offer herself as a PVS surrogate, before experiencing the event that causes her PVS. The likelihood of this ever happening is vanishingly small. So much so that Ber’s idea starts to look more like a thought experiment than a solution to a real-world problem.

My suggestion of using the organ donation framework means that (a) we have more potential candidates and (b) we have existing consent systems whereby people either give consent proactively in advance or are deemed to have done so in the lack of any evidence to the contrary. Thus, wherever organ donation is legal, brain-dead WBGD would be a relatively simple tweak to that framework.

However, the consent requirements for organ donation are extremely loose, in comparison with consents required for other forms of medical intervention. Recent legislative changes in the UK, for example, mean that a person’s organs may be harvested without any clear indication that they wished for this to happen. Should we expect something more demanding than this, if we include WBGD among the uses of a person’s body after their (brain) death? If so, why, given that we accept such minimal requirements for ‘normal’ organ donation? Perhaps one answer here is that WBGD is not something that people understand or have knowledge of. Therefore ‘deemed consent’ such as the organ donation framework relies on, is not properly informed. People who fail to opt out of the organ donation system can be regarded as having passively consented to something they have sufficient knowledge about. Everyone has heard of organ donation. No-one has heard of WBGD. Moreover, WBGD is qualitatively different in that it entails ventilation over an extended period. And, of course, its aim is not ‘life-saving’ per se as organ donation is usually understood to be.

In fact, the public is poorly informed as to the details of cadaveric organ donation and harvesting; some of those who support organ donation in principle might be disturbed if they understood what is involved, or even choose not to donate [18, 19]. Certainly, the level of information that is deemed sufficient as a basis for harvesting organs is minimal when compared with other significant invasive procedures either before or after death. Consenting to an operation would require a far greater degree of information; making a will would require a far greater degree of specificity and would need to be witnessed in order to be legally binding. If current consent protocols are acceptable for organ donation, they should be acceptable for WBGD, perhaps with additional public information campaigns.

## Extended ventilation

WBGD in brain stem dead patients would entail lengthy periods of ventilation. Some clinicians regard somatic survival after brain stem death as being unsustainable for prolonged periods. The UK’s National Health Service (NHS) states that “…the heart will eventually stop beating, even if a ventilator continues to be used” [20]. But this is not very helpful: any heart will eventually stop beating, ventilated or not. The question is when the heart will stop, and whether this can be controlled or postponed.

The maximum period for which a brain-dead patient can be somatically supported is unknown. Part of the reason for the NHS’ oddly worded statement above is to encourage relatives to accept that their loved one should not be ventilated indefinitely; to be able to ‘let go’. It is precisely *because* somatic function can be vastly prolonged that the NHS makes this statement. It is commonly regarded as bad medical practice, as well as being unethical, to prolong somatic survival in brain-dead patients. I suggest that at least one factor here relates to the discomfort that arises from the liminal state between life and death, that brain-dead patients occupy. Although clinically they are deemed ‘dead’ we find it hard to act as though this is really uncontrovertibly true, in comparison with cadavers, for example. Ventilated patients are warm and have a healthy colour from the circulating of their blood; cadavers are cold and discoloured. A cadaver will decompose quickly if not chemically preserved or refrigerated. The ventilated organ donor will not decompose unless some additional event or intervention occurs.

All of this makes healthcare providers reluctant to prolong this paradoxical state of death-in-life. And it can be hard for relatives too. However, brain-dead patients *can* be sustained for prolonged periods if we overcome our distaste for doing so. Of the documented cases of prolonged somatic survival (with and without gestation as a complicating factor), many end in death specifically because ventilator support was withdrawn–for ethical or legal reasons. For example, Abuhasna Said et al. note that the duration of the longest brain-dead gestation is 110 days. The foetus in this case was delivered at the earliest point at which it was deemed viable, at 32 weeks’ gestation. Ventilation was withdrawn from the mother immediately after delivery, resulting in her ‘death’ [7]. Such cases do not tell us how long the patient *could* have been sustained if ventilation had continued [21]. Sarah Armstrong and Roshan Fernando observe that there is no known upper physiological limit to the “prolongation of somatic function in the absence of brainstem function” [22].

Prolonging ventilation and somatic survival in brain-dead patients is undoubtedly a disturbing prospect. WBGD involves treating the patient’s dead body as a means to an end, rather than as an end in itself. The patient moves from being the focus of medical concern, to being a repository of tissues that can be used to benefit others. The prolongation of the ventilation period exacerbates our awareness of this. Yet this is already a part of our organ donation process. Organ donors are almost invariably patients who are already being ventilated, as part of their medical treatment. If the patient is deemed to be a suitable organ donor, ventilation will be continued along with other interventions to ensure that the organs will be maintained for transplant in optimal condition. Thus, we already prolong ventilation in order to facilitate organ donation.

WBGD would involve extending this prolongation considerably further. But ventilating someone for two days, two weeks, or two years makes little difference *except* insofar as it forces us to acknowledge and recognise what we are doing before we hasten on to the next stage. The justification for prolonging somatic survival in conventional organ donation is primarily the benefits that are expected to derive for others, but also the idea that if someone wants to donate their organs, it may be reasonable to take the steps to preserve the organs even when this is no longer directly in the patient’s medical best interests. The same criteria apply to WBGD; the period of prolongation is further extended, but the means and justification are the same.

There may be practical issues, however, since the longer period of ventilation required for WBGD would give scope for more medical complexities than those involved in conventional organ donation. Not only this, but there may be a question as to the feasibility of initiating pregnancy in brain-dead patients. There are at least two reported cases of PVS patients *becoming* pregnant, after their PVS diagnosis after being raped, as Ber reports [1]. But to date, there are to my knowledge no documented reports of the initiation of pregnancy in brain stem dead patients. This could mean that the incidence of rape in brain stem dead patients is zero, in contrast to that in PVS patients. Alternatively, it might suggest that the incidence of rape is similar in both cases, but that rape in brain-dead patients does not result in pregnancy. (It is perhaps misleading to use the term ‘rape’ in the case of brain-dead patients, if we really regard the victim as being dead. Sex with a corpse is necrophilia rather than rape.)

The ability of PVS patients to become pregnant indicates that PVS is not incompatible with normal hormonal and biological processes. Brain stem death involves a much more sweeping impact on the body’s normal functions. Blood pressure, temperature and hormonal balance all require artificial maintenance and monitoring in brain stem dead patients on ventilators. It may be unlikely that a patient could become pregnant in the ‘natural’ way without additional hormonal intervention. However, both in Ber’s argument and in my adapted proposal for WBGD, pregnancy need not arise through ‘natural’ conception. As with many surrogacy arrangements, commissioning parents may prefer to create an embryo for implantation using their own gametes or those of donors. Thus, impregnation could be a surgical affair, preceded and followed by appropriate hormonal therapy to ensure maximal chance of success. But even so, would it work?

Armstrong and Fernando note that the lowest gestational age at which a foetus can survive in a brain-dead mother has not yet been determined [22]. Said et al. go further in pointing out that with advances in critical care medicine, early gestational age of the foetus is no longer a limiting factor in terms of its prognosis [7]. However, up till recently, it has been regarded as inappropriate to prolong a brain-dead pregnancy in which the foetus was of less than 16 weeks’ gestational age. This cut-off point seems to have been the product of a combination of assumptions incorporating beliefs about the moral status of the pre-viable foetus as well as the likelihood of success. Again, therefore, there is a lack of data here that arises at least in part from uncertainty and moral squeamishness about the prospect of prolonging somatic survival of pregnant brain-dead women.

All those who discuss these issues agree that there is a lack of data. If WBGD has anything at all to recommend it, this gives us a prima facie reason at least for seeking additional information. We will not know what variables affect the outcomes without carrying out further research. But even without having undertaken such research it is evident that WBGD will offer some benefits over standard cases of brain-dead gestation as reported in the literature. Every case of brain-dead pregnancy reported to date involved a catastrophic event that happened to a woman *after* the initiation of her pregnancy. Whether through trauma, spontaneous haemorrhage or other causes, the woman and her foetus have already been adversely affected by the event that caused her brain death. Not only this, but women and their foetuses are often further damaged by aggressive attempts to save them. Case reports bear this out, detailing a catalogue of attempts and failures to save the patient [7]. The patient’s condition fluctuates as she goes through the transition from healthy pregnant woman to critically ill patient, to brain-dead patient.

The foetus, if it survives all of this, will also have undergone a significant trauma. On top of all this, the lack of experience and accumulated knowledge of how to manage brain-dead pregnancy have played a significant part in the fate of the foetuses involved in the cases so far reported. Notwithstanding this unpropitious start, it seems that those foetuses who do make it to delivery do well. David Powner et al. report on a range of cases in which pregnant brain-dead women’s somatic functioning was prolonged. They followed up the offspring and found that all seemed to be developing normally except one who was born with congenital abnormalities caused by the mother’s use of phenytoin (taken for epilepsy) [23].

WBGD would be likely to have better outcomes precisely because it would only be carried out in those patients in whom somatic support had been achieved and stabilised. Moreover, in the case of WBGD, since the pregnancy is deliberately initiated and the *primary aim* from its outset is the wellbeing and survival of the foetus, there would be no point at which the mother’s interests were presumed to be in conflict with those of the child. By contrast, in each of the reported cases to date, the decision to focus on trying to sustain the foetus was not made until some way through the mother’s treatment process when some therapies detrimental to the foetus would have been tried on the mother. Given all these considerations, it seems that there are grounds to think the prognosis of foetuses in a WBGD scenario would be better than those reported in the literature to date.

Given the current state of medical science, as outlined above, WBGD is not beyond the realms of possibility. Since we are happy to accept that organ donors are dead enough to donate, we should have no objections to WBGD on these grounds. WBGD donors are as dead as other donors – no more, no less. Since we are happy to prolong the somatic survival of already pregnant brain-dead women, to initiate pregnancy among eligible brain-dead donors should not trouble us unduly. But to move towards the actuality of WBGD, some further argument may be required to show why WBGD is ethically desirable, and to demonstrate why, in the face of the most obvious objections, it may nevertheless be a preferable alternative to uterus donation and surrogacy and even to pregnancy itself.

## The status of the WBGD embryo and foetus

Undeniably, in our present state of knowledge, much remains to be learnt about prolonged somatic survival, initiation of pregnancy, gestation, and delivery in brain-dead patients. Even those who might think there is some appeal in WBGD might baulk at the idea of how we could move ahead in the experimental phase that would be required before we could be sure that WBGD is safe and effective for routine use. This hiatus is not unusual. It exists between every prospective innovation, and our current practices. But in the case of WBGD, we face the problem of what it might mean to embark on experimental procedures that affect real embryos, foetuses and, ultimately, babies.

In jurisdictions that already permit embryo research, it is clear that some experiments on implantation and development up to 14 days might be permissible. Within the existing infrastructure of these jurisdictions, there seems little reason why preliminary experimentation should not go ahead. However, moving from experimental procedures designed to end in the destruction of the embryo at 14 days, to experiments that affect later stage foetuses, or which might be designed to result in the birth of live offspring, may be contentious. Nevertheless, it is worth noting that in recent years, the 14-day rule has started to come under some pressure both from scientists and ethicists who believe there should be a longer period during which research is permissible.[24].

Foetuses have greater protection than embryos in some jurisdictions. Harm, or even uncertainty relating to the foetus in utero as a research subject might therefore pose a problem. However, in places where embryo research is permitted, the law often allows for abortion. Legal grounds for abortion generally include impairments or diseases affecting the foetus. Thus, with very close surveillance, it is reasonable to think that–if foetuses are severely damaged by unexpected factors arising from brain-dead gestation–this need not result in the birth of severely damaged babies. Rather, it could result in the termination of the process at the discretion of the commissioning parents. Abortion, especially late term abortion, can be traumatic for gestating women both emotionally and physically. However, in the case of WBGD, the gestating woman is already dead and cannot be harmed. Commissioning parents may decide on abortion or selective reduction in accordance with their own wishes, without having to worry about the effects on the gestating donor.

This is an important consideration: abortion is one of the issues that make surrogacy ethically troublesome. Getting pregnant on behalf of a commissioning parent is one thing but being required to undergo an abortion seems to push the boundaries of what is acceptable in medicine, yet it is a fairly standard part of surrogacy contracts. In addition, surrogacy contracts often include clauses that require the surrogate to undergo or forego certain medical interventions. This may be construed as relinquishing a right that, properly speaking, is inalienable. In the case of WBGD, we face no such difficulties. As the gestational donor is in some ways much more explicitly the proxy of the commissioning parents, than a surrogate, it is not necessarily a stretch to regard selective reduction or the removal of a damaged foetus, as undergoing abortion by proxy.

In other examples of innovative fertility treatments whose effects on foetuses and offspring are uncertain, we accept that parents go ahead, hoping for the best, but recognising that in the event of a bad outcome, the pregnancy will be terminated. In some ways, WBGD offers a more familiar way forward than, for example, IVF when it was first undertaken in humans, and mitochondrial donation. It also offers a better-known path than uterine transplantation, whether living or cadaveric. We already know that human foetuses can survive gestation in brain-dead patients. This is more than Patrick Steptoe and Robert Edwards knew when they created the embryo that would become Louise Brown and more than Mats Brännström and his team knew when they brought about a pregnancy in a transplanted uterus [25].

Given that we already treat fertility medicine as an arena in which embryos and foetuses may be damaged or deliberately destroyed, it is not clear that the admitted uncertainties involved in WBGD are such as to force us to repudiate the whole endeavour. A final point here is that in fact WBGD offers a further benefit over standard pregnancies: the WBGD donor is under absolute medical control and surveillance. The move towards greater surveillance of pregnancy in living women has been strongly criticised by many feminists for its oppressive and intrusive incursions into the everyday lives that women must still live while pregnant. The WBG donor *has* no everyday life: her function is solely to gestate. We dare not transfer too many embryos into living women, because selective reduction is traumatic and harmful to the pregnant woman. There are no such problems in relation to the WBG donor. If she needs more or less of any particular drug or if foetal interventions are required, we have none of the potential conflict that can affect ordinary pregnancies. Parents may transfer as many embryos as they can generate, maximising the chances of at least one viable birth, and if necessary, discarding any damaged or diseased ones in advance. Again, pointing out these possibilities may sound ugly, but they are processes that are routine in fertility medicine across the globe.

## Who needs WBGD?

We are frequently told that people die while waiting for an organ transplant. By donating, then, we save lives that would otherwise be lost. In contrast, WBGD is not a life-saving intervention. Perhaps on this basis, we should focus on interventions where the clinical need is demonstrably greater. Yet although a heart or liver transplant may literally save someone’s life, many transplantable organs and tissues are not directly lifesaving. The corneas, even the kidneys, may improve the quality of a person’s life, and might increase one’s life span, but since people can live without eyes, and survive for many years with dialysis, the insistence that organ donation should be ‘life saving’ seems outdated. With increasing expertise in transplant surgery, the options for non-life-saving interventions–face, larynx, hand, uterus, and so on–are multiplying. If we accept this, we have no grounds to object to WBGD on the basis that it is not a life-saving intervention. Indeed, WBGD in some senses can be *more* accurately described as ‘helping someone to live’ than many other forms of donation, since it effectively allows for the creation of a new life.

Unlike any other form of organ donation, WBGD imposes no risks on the ‘recipient’. It has the additional advantage of conveying significant clinical *benefits* on women who make use of it. If WBGD were offered as an alternative to pregnancy generally, the clinical benefits would be striking. It is here that I diverge most significantly from Ber. Ber argues that only the neediest of claimants should have access to WBGD – those who have clear medical contra-indications to pregnancy or lack a uterus altogether. The problem with this is that pregnancy itself should properly speaking be medically contra-indicated for women generally.

It is well known that pregnancy and childbirth carry significant health risks, even in affluent settings with sophisticated healthcare systems [26, 27]. To expose oneself to risks comparable to pregnancy and childbirth would be deemed foolish and pathological in any other context. I have previously shown that in a comparison between pregnancy and measles, pregnancy comes out considerably the worse in terms of morbidity and mortality [28]. Yet concerted medical efforts are focussed on ridding ourselves of measles, while women are expected to submit themselves to the greater risks of pregnancy and childbirth almost without thinking about it. Measles is a notifiable disease whose eradication is an avowed goal of medicine. It follows that pregnancy should–all other things being equal–also be regarded in this light, since it is riskier than measles. We cannot yet forego the uterus altogether for the reproduction of our species. But we *can* transfer the risks of gestation to those who are no longer able to be harmed by them.

## Feminist concerns and male pregnancy

There are aspects of WBGD that might stand out as being unacceptable from a feminist perspective. WBGD clearly dissociates the functions of reproduction from the person. The reproductive capacity is in some senses commodified; it is valued for what it can produce rather than its intrinsic association with the person whose capacity it is. Women are often objectified for their sexual or reproductive functions, even while they are very clearly alive. The idea that a pregnant woman is, or should be treated as, a foetal container, frequently reasserts itself [29]. WBGD *is* quite straightforwardly the use of the body as a foetal container. Could it be that in allowing such use, we would somehow condone the idea that living women who are gestating are also to be treated as mere foetal containers?

One might argue that WBGD involving brain-dead women has no implications for living women, any more than harvesting the heart from a brain-dead man has an impact on living men. However, perhaps this is disingenuous. WBGD necessarily involves the separation of women’s reproductive functions from their very consciousness. Even if no-one would suggest that this should alter the way we regard ordinary women and their pregnancies, it might send an implicit message, or reinforcement to deeply entrenched assumptions and prejudices. The prospect of the unconscious woman’s body, filled and used by others as a vessel, is a vivid illustration of just what feminists have fought against for many years.

These feminist concerns, however, might be mitigated if men could also participate in WBGD. The prospect of male pregnancy is not, as many would imagine, fanciful, or a piece of science fiction. In 1999, Robert Winston told reporters that there were no intrinsic medical problems with initiating a male pregnancy: the danger would be in the delivery. We already know that pregnancies can come to term outside the uterus [31]. The liver is a promising implantation site, because of its excellent blood supply. However, as Winston noted, this could be risky – even fatal - for the person carrying the pregnancy. But for brain-dead donors, the concept ‘fatal’ is meaningless: the gestator is already dead. Thus, even if the liver is damaged beyond repair after the gestation, this would not pose a problem except insofar as it might mean that male gestators could carry only one pregnancy, rather than many consecutive ones.

The prospect of the male gestator could thus appease some feminists who might otherwise feel that brain-dead gestation is a step too far in the objectification of women’s reproductive functions.

## Conclusion

Rosalie Ber’s idea of using women in PVS as substitutes for surrogates has received surprisingly little attention since she first published her paper. My adaptation of her suggestion would enable more people to donate, and more people to benefit. It requires no redefinition of concepts such as brain death or PVS. For these reasons, WBGD deserves serious consideration. Of course, this proposal may seem shocking to some people. Nevertheless, as I have shown, if we accept that our current approach to organ donation and reproductive medicine are sound, WBGD donation seems to follow relatively smoothly from procedures that we are already undertaking separately. What I put forward here can be viewed as a thought experiment on one hand. But if we regard WBGD as being clearly outrageous, this suggests we have some uncomfortable questions to answer about the future of cadaveric organ donation.

On the other hand, if WBGD is viewed as a straightforward means of facilitating safer reproduction, and avoiding the moral problems of surrogacy, we should be ready to embrace it as a logical and beneficial extension of activities that we already treat as being morally unproblematic.
